# Reliability of radial arterial pressure monitoring after cardiac surgery

**DOI:** 10.1186/cc10821

**Published:** 2012-03-20

**Authors:** C Lavault, MC Fevre, A Hebrard, M Durand, F Grimbert, Y Lavault, P Albaladejo

**Affiliations:** 1 UJF - Grenoble1/CNRS/TIMC-IMAG UMR 5525 (Equipe PRETA), Grenoble, France; 2 CHU Grenoble, France

## Introduction

Invasive monitoring in critically ill patients allows a continuous measurement of arterial pressure, cardiac output, and the derivation of dynamic predictors of fluid responsiveness. However, the pressure signal may be altered by the dynamic characteristics of the fluid-filled tubing. The aim of the present study was to evaluate the reliability of radial artery blood pressure measurement and derived indexes during the early period after cardiac surgery.

## Methods

After IRB approval, 30 patients admitted to the ICU after elective cardiac surgery (CABG: 16, valve surgery: 11; combined: 3) with a radial artery catheter were included. In the ICU, an independent continuous recording of arterial pressure during at least 18 hours was started via a double-head pressure transducer (Flotrac; Edwards Lifesciences, Irvine, CA, USA) for a retrospective analysis and three fast flushes were performed. First, the whole record was examined for episodes of overdamping (Ov) or attenuation (At). Ov was defined as a decrease in systolic (sAP), an increase diastolic (dAP), and an unchanged mean pressure (mAP). At was defined as a decrease in sAP, dAP and mAP. Second, three periods of 10 minutes during the first hour were analysed assuming that the dynamic characteristics remained constant. This allowed the correction of the distorted raw signals and the study of the consequences of an underdamped signal on sAP, pulse pressure variation (PPV) and dP/dt as an estimate of left ventricular contractility. A paired *t *test was used for statistical comparison, *P *< 0.05 was considered statistically significant.

## Results

Mean age was 69 ± 13 years, 14 patients received noradrenaline, eight patients dobutamine, and nine patients volume expansion. During the whole record, the number of episodes of Ov or At ranged from 0 to 15 with a duration of 0 to 6 hours: 17 patients had at least one episode of Ov and/or At tracing, 10 patients had at least two episodes, eight patients had at least five episodes. Seven episodes lasted more than 20 minutes and three more than 1 hour. During the first hour, sAP was overestimated by 5.0 ± 1.4 mmHg (*P *< 0.0001) (range: 0.3 to 5.9) or by 4.3 ± 0.9% (range: 0.4 to 15.9%), raw PPV was 9.5 ± 7.3 versus 10.0 ± 7.8 for the corrected PPV (range from -2.6 to 4.3); raw dP/dt was overestimated by 134 ± 47 mmHg/second (*P *< 0.0001) (range: -13 to 353) or by 24 ± 6%.

## Conclusion

These results showed that frequent artefacts and distortions induced by the fluid-filled tubing could modify the arterial waveform and could lead to inaccurate therapy [1]. More attention should be paid to the quality of the pressure signal.
